# Quality of life, spiritual needs, and well-being of people affected by infertility and its treatment: quantitative results of a mixed-methods study

**DOI:** 10.1007/s10815-025-03463-z

**Published:** 2025-04-01

**Authors:** Madeleine Bernet, Alexander M. Quaas, Michael von Wolff, Alessandro Santi, Isabelle Streuli, Dorothea Wunder, Eva Soom Ammann, Arndt Büssing

**Affiliations:** 1 Division of Nursing, Department of Health Professions, Bern University of Applied Sciences, Bern, Switzerland; 2 Faculty of Health, Witten/Herdecke University, Witten, Germany; 3 Shady Grove Fertility, San Diego, USA; 4 Division of Gynaecological Endocrinology and Reproductive Medicine, University Women’S Hospital, Inselspital, University Hospital, Berne, Switzerland; 5 Ospedale Regionale di Locarno, Locarno, Switzerland; 6 Division of reproductive medicine and gynaecological endocrinology, Hôpitaux Universitaires de Genève, Geneva, Switzerland; 7 Hôpital Cantonal Fribourg, Fribourg, Switzerland

**Keywords:** Infertility, Quality of life, Well-being, Quality of care, Spiritual needs, Healthcare

## Abstract

**Purpose:**

Infertility is a growing global health issue that significantly affects quality of life (QoL). Understanding its impact on QoL is essential for developing effective healthcare interventions. This study explored QoL, well-being, and spiritual needs among individuals affected by infertility, with implications for healthcare provision.

**Methods:**

Standardized questionnaire data from an anonymous mixed-methods study that was conducted from October 2022 to January 2023 in Switzerland and addressing QoL (FertiQoL), spiritual needs (SpNQ-20), and psychological well-being (WHO-5) of individuals undergoing fertility treatments.

**Results:**

The analysis included 326 participants. FertiQoL scores were lowest in the emotional domain (*M* = 46.35) and highest in the relational domain (*M* = 68.51), with a mean overall score of 56.69, indicating moderate QoL impacts. Participants without children reported significantly lower FertiQoL and WHO-5 well-being scores than those with children (*p* < 0.05). The WHO-5 mean score indicated moderate well-being (*M* = 13.89). SpNQ-20 results showed the highest needs in inner peace (*M* = 1.82), followed by generativity (*M* = 1.09) and existential needs (*M* = 0.86), with religious needs scoring the lowest (*M* = 0.43). Participants without children had significantly higher spiritual needs (*p* < 0.05).

**Conclusion:**

This study highlights the emotional and spiritual challenges of infertility, revealing differences in experiences between individuals with and without children. It emphasizes the importance of addressing mental health and well-being in infertility care. Further research should focus on the psychological impacts of fertility treatments, including depressive mood states.

**Supplementary Information:**

The online version contains supplementary material available at 10.1007/s10815-025-03463-z.

## Introduction

Infertility is an increasingly recognized global health issue affecting millions of people of reproductive age worldwide. Available data suggests that globally, one in six people experiences infertility in their lifetime [1]. Since 1993, Switzerland has documented data on assisted reproductive methods in the FIVNAT registry, and in 2022, 3% of live births resulted from in vitro* fertilization* (IVF) [2].

Infertility can significantly impact an individual’s QoL and encompasses both physical health and emotional well-being, social functioning, and a person’s beliefs and relationship to their environment [1]. Fertility treatment can dominate the daily routine and couples navigating fertility treatment commonly contend with emotions including hope, optimism, disappointment, sadness, isolation, guilt, denial, and non-acceptance [3, 4]. Among the many challenging phases, the wait between embryo transfer and the subsequent pregnancy test is particularly emotionally taxing for all people involved [3]. Unsuccessful fertility treatment can cause feelings of loss and grief, and women have an especially increased risk of developing depression [4–6]. However, women who proactively seek social support, mentally prepare for the possibility of treatment failure, and recognize their emotional challenges tend to exhibit fewer depressive signs [7], suggesting that awareness and reflection of the individual situation might be important for maintaining mental health and resilience during this stressful period of life.

Spirituality refers to peoples’ search for meaning (immanence) and for the sacred in life (transcendence). In times of illness, it can help explain what has happened [8]. Infertility—when seen as a crisis in life—can cause people to reflect deeply on the meaning and purpose of life. Numerous studies suggest that many patients, whether religious or non-religious, find comfort in their underlying spirituality as a resource for finding meaning, hope, and direction in life [9–12]. Therefore, health professionals need to empathize with patients and consider their spiritual well-being [13]. Although many studies investigating the psychosocial impact of infertility have been performed, reliable and representative data on the impact of infertility and infertility treatment on mental health and spiritual needs is lacking. This study aimed to analyze the current situation in the field of reproductive medicine in Switzerland from the perspective of affected individuals. It aimed to explore different indicators of well-being and the spiritual needs related to infertility and infertility treatment.

## Material and methods

### The HoPE study

The health care users’ and professionals’ perspectives and experiences in fertility treatment in Switzerland (HoPE) study [14] is a mixed-method study with both quantitative and qualitative components, utilizing an explanatory sequential design as outlined by Creswell and Plano Clark [15]. The results of the quantitative part of the study are presented in this manuscript. The qualitative component of the study, including interviews and focus groups with healthcare professionals and patients, will be published elsewhere.

Quantitative data were collected via an online survey between October 2022 and January 2023 and analyzed thereafter. The survey utilized three validated instruments: the Fertility Quality of Life tool (FertiQoL) [16], assessing emotional, mind–body, relational, and social dimensions; the WHO-5 Well-Being Index [17], measuring well-being over the past 14 days; and the Spiritual Needs Questionnaire (SpNQ-20) [13], addressing existential, religious, generativity, and inner peace needs in terms of their presence and intensity. A total of 326 individuals participated.

### Recruitment and sample

The recruitment process was designed to reach a diverse cohort of individuals with fertility problems. Flyers with QR codes and posters were actively distributed through partner institutions, medical practices, and associations throughout Switzerland. The Bern University of Applied Sciences promoted the study on its social media platforms between October 2022 and January 2023. The social media outreach proved to be the most effective, with approximately 75% of participants reporting that they have heard about the project through social media channels. The study was open to individuals who were currently undergoing fertility treatment (in Switzerland or abroad) or who had undergone such treatment in the past 5 years. Other inclusion criteria were age ≥ 18 years, residence in Switzerland, fluency in German, French, or Italian, and the physical and psychological ability to complete an online survey. For this study, a convenience sampling approach was used to include all eligible individuals who completed the questionnaire. The target sample size was a minimum of 120 completed questionnaires. A 5% margin of error was chosen to ensure the accuracy and reliability of our results.

### Data collection

Data on individuals who were undergoing or had undergone fertility treatments in the last 5 years were collected by an anonymous online survey from October 2022 to January 2023. Components of the survey included standardized measures such as fertility-related quality of life (FertiQoL) [16], spiritual needs (SpNQ-20) [13], and psychological well-being (WHO-5) [17], as well as sociodemographic details. For the research data collection, all instruments were applied in German, French, and Italian languages, representing Switzerland’s diverse language regions. Sociodemographic data and other information were translated by researchers proficient in the relevant native languages and subsequently reviewed by the co-authors.

To evaluate the impact of fertility problems on overall QoL, the FertiQoL (range: 0–100, with higher scores indicating better QoL) was applied which is a multidimensional measure that addresses the impact of fertility issues on health, emotions, self-perception, relationships, social dynamics, career, goals, and satisfaction with treatments [16]. The FertiQoL consists of 36 items of six subscales and three total scores. This allows to evaluate emotional, mind–body, relational, and social aspects of FertiQoL and addresses feelings, physical and cognitive health, and social interactions. In addition, the environment and tolerability of fertility treatments can be assessed, focusing on access, quality, and impact on daily life. The total FertiQoL score integrates both modules for an overall fertility-related QoL measure. Internal consistency was confirmed by calculating the Cronbach’s alpha coefficient for the FertiQoL questionnaire used in this study. The value of 0.86 was in line with previous reports stating Cronbach’s alpha coefficients of 0.82–0.93 [18].

The SpNQ-20 was used to assess the psychosocial, existential, and spiritual needs of the participants. The Cronbach’s alpha coefficient calculated for the SpNQ-20 questionnaire used in this study was 0.88, confirming good internal consistency which has also been reported in previous studies [13]. It differentiates religious, existential, inner peace, and generativity needs. The participants rate whether they currently have the respective needs and how strong they were to them. To measure the significance of spiritual needs for the individual, the instrument uses a 4-point scale from disagreement to agreement (0—not at all; 1—somewhat; 2—strong; 3—very strong).

To address the overall psychological well-being of the participants, the WHO-5 Well-Being Index [17] was utilized, consisting of five questions that evaluate well-being over the past 2 weeks. The raw score ranges from 0 to 25, with 0 representing the worst possible and 25 the best possible well-being. Scores < 13 could indicate depressive mood states. To obtain a percentage score ranging from 0 to 100, the raw score is multiplied by 4. The Cronbach’s alpha coefficient calculated for the WHO-5 questionnaire used in this study was 0.83, indicating good internal consistency.

### Data analysis

Descriptive data analysis was performed using standard descriptive statistical methods. For the comparison of binary subgroups, such as the presence or absence of children, we conducted independent-samples t-tests assumption of normality was met or Mann–Whitney U tests. The significance level was set at *p* < 0.05 for all analyses. All statistical analyses were performed using IBM SPSS 28.

### Ethical considerations

The study was evaluated by the Cantonal Ethics Committee of Bern (BASEC-No: Req-2021–00532). The Ethics Committee stated that they had no jurisdiction over the study as it does not fall under the scope of the Human Research Act, Art. 2, Para. 1. Additionally, an ethics application was submitted to the Ethics Committee of the University of Witten/Herdecke (Nr. S-114/2022). No ethical or professional reservations were identified regarding the project. Participation was entirely voluntary and did not affect any current medical treatments that participants might have been receiving during the study period.

## Results

### Description of study participants

In total, 372 individuals were recruited who were either currently undergoing or had undergone infertility treatments in the past 5 years. Thirty-five people did not meet the inclusion criteria and were removed from the sample, resulting in a final sample size of 337 participants. Due to a low male participation rate (*n* = 11), the analysis focused exclusively on the 326 female respondents. Most of the participants were married or in a registered relationship (71.5%, *n* = 233), 25.8% were in a solid partnership (*n* = 84), 2.1% were single (*n* = 7), and 0.6% were engaged (*n* = 2).

The questionnaire was filled out in either of three languages: German (75.2%, n = 245), French (18.4%, n = 60), or Italian (6.4%, *n* = 21). The distribution of responses is broadly reflective of the linguistic landscape of Switzerland (62.3% German, 22.8% French, and 8% Italian) [19].

The mean age of all participants was 36.8 years (SD 4.35), and half of them had children during the study period (50.3%, *n* = 164). The average duration of infertility in the study cohort was 21.84 months (SD 20.25). Most of the participants (73.3%, *n* = 239) were covered by basic insurance, with the remaining 26.7% (*n* = 87) participants having supplementary insurance. Concerns about their financial situation were expressed by 50.9% (*n* = 166) of respondents, despite 36.2% (*n* = 118) and 27.3% (*n* = 89) reporting an annual household budget exceeding 100,000 CHF and 150,000 CHF, respectively—amounts that are above the average in Switzerland.

Assisted Reproductive Technology (ART), i.e., IVF and intracytoplasmic sperm injection (ICSI), was the most common treatment method reported (66.3%, *n* = 216). A total of 113 participants (43.6%) underwent five or more fertility treatment cycles. The majority received treatments in Switzerland, while about one-fifth sought treatments abroad (21.2%, *n* = 69). Thereof, 26 participants (8%) had undergone egg donation, 4.9% (*n* = 16) sperm donation, 1% (*n* = 3) embryo donation, and 2.8% (*n* = 9) had done treatment using a gestational carrier. A total of 136 study participants (41.7%) experienced one or more miscarriages. More detailed information on the study participants can be found in Supplementary Table I.

### Results FertiQoL—dimensions

**Fig. 1:**
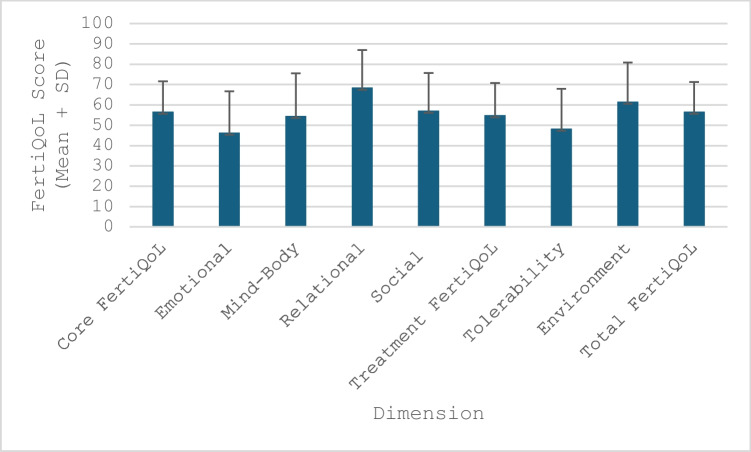
FertiQoL-scores. FertiQol range = 0-100, whereof a score of 100 indicates maximal positive impact of fertility issues on health, emotions, self-perception, relationships, social dynamics, career, goals, and satisfaction with treatments. The items belonging to the respective subscale are indicated in brackets

In total, 304 participants filled out the FertiQoL core module, and participants currently undergoing treatment additionally filled out the optional treatment module (*n* = 109). The mean score measured for the emotional subscale (46.35) was low, followed by the mind–body (54.56) and social dimension (57.12), while the relational dimension received the highest rating (68.51). In the treatment module, a lower mean score was observed for the tolerability dimension (48.34) than for the environment dimension (61.61). The scores for the individual dimensions and modules and the total FertiQoL score are presented in Fig. 1.

### Results FertiQoL—on item level

Participants rated their physical health highly, with 57.2% reporting “good” and 27.3% “very good.” Satisfaction with general QoL was also high, with 65.5% feeling “satisfied” and 15.8% “very satisfied.” In total, 48% felt moderately able to manage fertility-related challenges, while over 60% frequently experienced feelings of jealousy and resentment. Additionally, 34.5% reported grief and/or loss related to infertility, and 40.5% experienced frequent fluctuations between hope and despair, with 15.1% feeling this consistently. Emotional distress was common with 36.3% and 29.4% feeling sad or depressed often and regularly, respectively. Anger was experienced often by 28.3% and frequently by 26.3%.

Fertility issues also impacted cognitive and physical functioning. Significant impairments in attention and concentration were reported, with 23.7% experiencing this “very often” and 39.5% “quite often.” Two-thirds of participants felt their fertility struggles hindered other life goals “very often” (29.6%) and “quite often” (30.6%), and 65% reported feeling drained or worn out due to infertility “quite often” or “very often” and 15% “always.” Fatigue was prevalent with 37% feeling “very often” and 22% “quite often” bothered by it. Regarding physical discomfort, 38.8% reported “never” feeling pain, while over 40% experienced discomfort “seldom” or “quite often.”

Socially, over 50% were satisfied with the support from friends, but 19.4% and 13.2% felt isolated “quite often” and “very often.” However, 42.4% indicated feeling socially isolated only “seldomly.” A total of 27.3% felt understood by their family, but 41.1% and 14.8% felt understood “seldomly” and “never.” Half of the participants felt inferior to those with children, and 27.0% felt social pressure to have children “very often,” 23.4% “quite often,” and more than 10% “always.”

In the optional treatment module, 75% reported being “moderately” to “extremely” bothered by the impact of fertility treatment on daily life. Most found the complexity of procedures and medications “moderately” (38.5%) to “very much” (21.1%) and “an extreme amount” (9.2%) challenging. Side effects were a concern, with 36.4% feeling “moderately” and 27.1% “very much” bothered. Satisfaction with emotional support services was mixed, with 40% satisfied, but over a quarter dissatisfied. The quality of information received was rated positively by 42.2%, while 20% found it poor. Interactions with medical staff were largely positive, with 67.8% either “satisfied” or “very satisfied.” Detailed findings can be found in Supplementary Table II.

### Results SpNQ-20

**Fig. 2:**
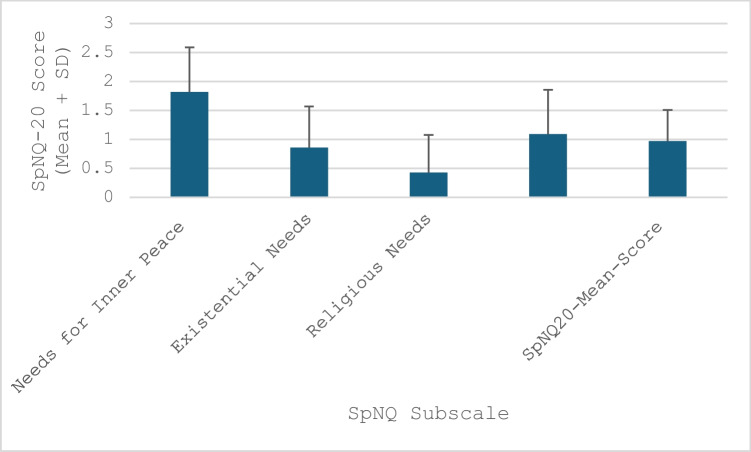
Results SpNQ-20 and scoring. SpNQ20 range = 0-3, whereof the highest score of 3 indicates a large need for psychosocial, existential, and spiritual, as well as for religious, existential, inner peace, and generative aspects

Important aspects for the participants were finding inner peace, believing in a higher presence, the need for forgiveness because of life reflection, and passing own life experiences to others, whereas talking with others about meaning in life and giving away something from themselves was less important. With respect to the SpNQ-20 subscale scores, participants scored high on the inner peace needs (1.82), moderate for generativity needs (1.09), low on existential needs (0.86), and lowest for religious needs (0.43). The scores are presented in Fig. 2, while detailed findings for the individual questions can be found in Supplementary Table III.

### Results WHO-5

In total, 293 participants completed the WHO-5 questionnaire. More than half of the respondents (52.9%) reported feeling cheerful and in good spirits “most of the time” or “always,” indicating a prevalent sense of positivity. Less (37.8%) felt calm and relaxed “most of the time” or “always,” suggesting a significant degree of emotional tranquillity among over a third of the study cohort. The participants reported feeling active and vigorous “most of the time” or “always” (23.7%), suggesting a moderate level of physical and mental energy in this group. Furthermore, a substantial number of participants reported waking up feeling fresh and rested more than half of the time or more often (43.7%). However, many respondents did not feel fresh and rested upon waking up most of the time (48.8%), and some never felt rested (8.5%). The life of most participants was filled with fulfilling daily activities (73.4%), reflecting a substantial level of satisfaction with daily routines. The mean sum score of the WHO-5 questionnaire was 13.89. Detailed information on the results of the individual questions of the WHO-5 are listed in Table 1.

**Table 1:** Results study HoPE WHO-5 Well-Being Index

Over the last 2 weeks	*n* (%)	*n* (%)	*n* (%)	*n* (%)	*n* (%)	*n* (%)
	All the time	Most of the time	More than half of the time	Less than half of the time	Some of the time	At no time
I have felt cheerful and in good spirits. (*n* = 293)	6 (2.0)	149 (50.9)	66 (22.5)	38 (13.0)	32 (10.9)	2 (0.7)
I have felt calm and relaxed. (*n* = 291)	4 (1.4)	106 (36.4)	78 (26.8)	49 (16.8)	48 (16.5)	6 (2.1)
I have felt active and vigorous. (*n* = 291)	3 (1.0)	66 (22.7)	82 (28.2)	72 (24.7)	61 (21.0)	7 (2.4)
I woke up feeling fresh and rested. (*n* = 293)	4 (1.4)	67 (22.9)	54 (18.4)	81 (27.6)	62 (21.2)	25 (8.5)
My daily life has been filled with things that interest me. (*n* = 293)	14 (4.8)	126 (43.0)	75 (25.6)	36 (12.3)	39 (13.3)	3 (1.0)
WHO-5-sum-score	13.89 (*n* = 293, SD:4.78, min. 0, max. 25)					
WHO-5–100-sum-score	55.56 (*n* = 293, SD:19.13, min. 0, max. 96)					

###  Well-being and spiritual needs of women with and without children

Indicators of well-being and spiritual needs related to infertility were assessed using the FertiQoL, WHO-5, and SpNQ-20. In the SpNQ-20 measure, individuals without children reported significantly higher mean scores, indicating greater spiritual needs compared to those with children (*p* = 0.003). For WHO-5 well-being indicators, women without children scored significantly lower than those with children (*p* = 0.002). Women with children reported significantly higher scores in the mind–body (*p* < 0.001) and emotional (*p* < 0.001) dimensions of FertiQoL. Women without children had significantly higher scores in the relational dimension (*p* = 0.006). Inner peace scores were significantly higher in women without children (*p* < 0.001). All significant differences showed small effect sizes (*η*^2^ < 0.06). Inner peace (*η*^2^ = 0.059) exhibited the largest effect size among the assessed variables. Table 2 presents the mean values (M) and standard deviations (SD) for various dimensions of fertility-related quality of life (FertiQoL), well-being (WHO-5), and spiritual needs (SpNQ-20) for women with and without children.

**Table 2:** Comparison of mean values of different parameters between women with and without children

		FertiQoL										WHO5	SpNQ-20				
		Mind–body	Emotional	Relational	Social	Environment	Tolerability	FertiQoL total	Core FertiQoL	Treatment FertiQoL	Well-being		Religious needs	existential Needs	Inner peace	Giving/ generativity	SpNQ20 mean scores
*n*		304	304	304	304	109	109	304	304	109	293		279	279	279	279	279
All women	M	54.56	46.36	68.51	57.12	61.61	48.34	56.69	56.64	54.97	2.79		0.43	0.79	1.62	1.07	0.89
	SD	21.05	20.32	18.44	18.50	19.27	19.56	14.52	14.98	15.79	0.96		0.65	0.74	0.79	0.80	0.58
With children	M	59.25	51.10	65.52	60.22	58.57	55.36	58.83	59.02	56.96	2.98		0.38	0.86	1.82	1.09	0.97
	SD	21.34	20.99	18.48	18.36	16.79	19.29	14.85	15.19	12.32	0.86		0.61	0.71	0.77	0.77	0.54
Without children	M	50.28	42.03	71.24	54.29	62.33	46.66	54.74	54.46	54.50	2.61		0.47	0.92	2.00	1.10	1.04
	SD	19.90	18.74	18.04	18.23	19.84	19.35	13.99	14.50	16.54	1.01		0.68	0.68	0.72	0.75	0.48
Eta2-value		0.045	0.050	0.024	0.026	0.006	0.031	0.020	0.023	0.004	0.038		0.004	0.009	0.059	0.000	0.019
*p*-value (*t*-test)		** < 0.001**			**0.005**	0.38	0.073	0.014	**0.008**	0.48			0.275				
*p*-value (Mann–Whitney U)		** < 0.001**	** < 0.001**	**0.006**	**0.008**	0.372	0.084	**0.026**	**0.016**	0.521	**0.002**		0.213	**0.031**	** < 0.001**	0.617	**0.003**

Eta^2^ values < 0.06 are considered as small effects, between 0.06 and 0.14 as moderate, and > 0.14 as strong; significant data are highlighted in bold

## Discussion

The impact of fertility on spiritual needs, measured with the SpNQ, represents a novelty of this study. The findings highlight the stress and challenges in dealing with fertility issues and uncover differences in the fertility experience between patients with and without children. In general, individuals without children tended to report higher levels of spiritual needs, inner peace, and existential fulfilment, potentially reflecting distinct lifestyle or psychological differences based on parental status. Individuals with secondary infertility reported greater overall well-being and satisfaction across emotional, relational, and social domains of fertility-related quality of life.

### Quality of life

The FertiQoL assessment highlighted the significant impact of fertility issues on individuals’ lives in Switzerland. Specifically, the emotional subscale had a low mean score of 46.35, highlighting the significant emotional burden. This finding aligns with previously published studies [3, 16, 20, 21] from other countries. Recent studies, including one conducted in a Dutch population, have revealed a significant correlation between core FertiQoL scores and established metrics for anxiety and depression [22]. The high risk for depression and anxiety in infertile women has also been shown in a meta-analysis by Almutawa et al. [23]. Ni et al. [5] reported a negative correlation of the fertility-related quality of life with the number of IVF cycles, accompanied by an increased risk of anxiety and depression. This is consistent with our finding of decreased emotional scores after four IVF cycles, suggesting a greater need for emotional support in those with multiple treatment cycles.

Cross-national comparisons of FertiQoL scores point to variability due to the differences in subscale assessments, treatment types, and sample characteristics [24]. Studies from the Netherlands, Japan, Spain, and Turkey reported higher FertiQoL scores compared to the HoPE study, but inconsistencies in assessment criteria and timing make direct comparisons challenging. In addition, gender ratios, typically female-dominant, and the restrictions on treatment types and infertility conditions might bias the results of different studies including ours. Additionally, financial factors, such as the high costs of treatments in Switzerland, may influence results. The diversity in study criteria underscores the complexity of interpreting FertiQoL scores across different populations.

### Spirituality

An important novelty of this study was the assessment of the impact of infertility on spiritual needs, using the SpNQ-20. The mean score for generativity needs was slightly above average, reflecting a moderate inclination towards contributing to the well-being of others, imparting wisdom, or leaving a lasting legacy. The overall mean score across all spiritual need categories was 0.97, indicating a broad range of spiritual needs, with a particular focus on inner peace and generativity, and less emphasis on religious and existential concerns.

More than half of the women expressed a desire to discuss their fears and concerns about infertility. This study is the first to assess spiritual needs in individuals with fertility problems using the SpNQ-20 and is limited to women, so future research should include both genders for a broader perspective. There were also regional differences, with Italian-speaking individuals reporting higher spiritual needs than French- or German-speaking individuals, possibly due to differences in religious and cultural affiliation [19].

The mean score for the need for inner peace was 1.82, indicating its importance to participants, while existential needs scored lower (0.86), showing that concerns about life’s meaning were less prominent. Religious needs had the lowest mean score, suggesting that most participants were less focused on religious rituals. This could reflect diverse religious affiliations or a less religiously inclined demographic. Religion plays a significant role in infertility treatments, influencing perceptions, decisions, and emotional well-being [25], as beliefs about medical procedures like IVF vary across cultures [26–28]. Additionally, religious communities can offer support networks for those facing infertility. Therefore, the impact of religion on fertility and QoL should be carefully considered.

### Exploring indicators of well-being and spiritual needs related to infertility

Infertility and the lack of children are strongly associated with lower overall well-being. Women without children reported lower scores in the mind–body and emotional domains (*p* < 0.001), reflecting significant physical and emotional challenges, in accordance with existing literature. [29–31]. Conversely, women without children scored higher in relational aspects of FertiQoL (*p* = 0.006), indicating a stronger relationship and bond to their partners. The lower social quality of life scores for women without children (*p* = 0.005) highlight the potential social isolation and lack of societal support experienced by those who are infertile. In addition, women without children exhibit higher spiritual needs, particularly inner peace needs (*p* < 0.001), suggesting that spirituality may serve as a crucial coping mechanism for women dealing with infertility. Our study is representative for a secularized society with both Protestant and Catholic roots, but also for a post-migrant society with a high degree of heterogeneity in terms of socio-cultural and therefore religious backgrounds. Previous studies have also noted that spirituality and religious coping can provide emotional support and resilience for individuals facing infertility [11, 32].

Our findings underscore the importance of improved support systems for women facing infertility that address and include emotional and spiritual dimensions in addition to physical dimensions as highlighted by Buran and Toptaş Acar and Shah et al. [3, 4]. Healthcare providers should consider incorporating psychological counseling and spiritual support into fertility treatments to help women navigate the emotional and existential challenges of infertility.

### Strengths and limitations

One of the key methodological strengths of our research was the use of tested and validated instruments. The inclusion of participants from the three language regions of Switzerland is a significant strength, as it reflects the cultural and linguistic diversity of the country and increases the generalizability of the results. However, our study also has limitations. Since the participants predominantly had a high level of education and household budget, they may not accurately reflect the entire general population affected by infertility. Participants also completed the questionnaires at various stages of their infertility journey, and not at one standardized treatment point, such as the window between embryo transfer and pregnancy test. Another limitation is the tendency of many responses to be moderate. Moreover, the low response rate and subsequent exclusion of men from the analysis limits the study to only female participants. Men were explicitly addressed during recruitment, but unfortunately, very few participated in the study. Furthermore, two of our survey tools, WHO-5 and SpNQ-20, were not developed specifically for an infertility population. Lastly, our study did not include a control group.

Direct comparisons between infertile individuals and the general population may be challenging due to the unique emotional and medical burdens of infertility. However, prior studies applying the same assessment tools to populations with chronic illnesses suggest a similar impact on the quality of life as well as psychosocial, existential, and spiritual needs of affected individuals. For instance, the SpNQ-20 has been used in patients with cancer, chronic pain, psychiatric conditions, and other chronic diseases, revealing that their highest-rated needs often relate to inner peace and generativity, while existential and religious needs vary based on cultural and personal beliefs [33].

### Recommendation for practice and future directions

The current study provides initial insights into the psychological consequences including QoL, spirituality, and well-being of individuals with fertility problems. The results clearly indicate a significant emotional burden, which should receive increased attention in the future. Professionally trained health care personnel could play a crucial role in enhancing comprehensive and individualized care, for example, by integrating questions from the validated instruments in this study into the standard patient assessment. We are currently continuing the HoPE mixed-methods study aiming for a broader participation of men and collecting data on the perspective of health care professionals. Future research should focus on identified long-term psychological burdens and consequences of infertility and include groups underrepresented in our study. In addition, future study designs should continue to tease out distinct aspects of women with and without children to generate individualized emotional resources for patients to assist with their fertility journeys.

## Supplementary Information

Below is the link to the electronic supplementary material.

ESM 1 (DOCX 52.1 KB)

## Data Availability

The data and materials of this study are available from the corresponding author, MB, upon request.
