# Supplementary material for: Quality of life, spiritual needs, and well-being of people affected by infertility and its treatment: quantitative results of a mixed-methods study
Source: J Assist Reprod Genet. 2025 Apr 1;42(6):1853–62. doi: 10.1007/s10815-025-03463-z (PMC12229361; doi:10.1007/s10815-025-03463-z)
Supplement: Supplementary file 1 — (DOCX 52.1 KB) [file 10815_2025_3463_MOESM1_ESM.docx]

**Appendix- Supplementary Tables**

Supplementary Table I: Characteristics of study participants

|  | **Number (n)** | **Percentage (%)** |
| --- | --- | --- |
| **Total number of participants** | 326 | 100 |
| **Mean age** |  |  |
|  | 36.8 |  |
| **Gender** |  |  |
| Female | 326 | 100 |
| Male | 0 | 0 |
| **Language** |  |  |
| German | 245 | 75.2 |
| French | 60 | 18.4 |
| Italian | 21 | 6.4 |
| **Already have children** |  |  |
| Yes | 164 | 50.3 |
| No | 162 | 49.7 |
| **Type of insurance** |  |  |
| Basic | 239 | 73.3 |
| Supplementary | 87 | 26.7 |
| **Annual household budget** |  |  |
| < 100.000 CHF | 119 | 36.5 |
| > 100.000 CHF | 118 | 36.2 |
| > 150.000 CHF | 89 | 27.3 |
| **Relationship status** |  |  |
| Single | 7 | 2.1 |
| Married | 233 | 71.5 |
| In partnership | 84 | 25.8 |
| Other | 2 | 0.6 |
| **Education** |  |  |
| Apprenticeship | 49 | 15.0 |
| University degree | 167 | 51.2 |
| Higher education | 104 | 31.9 |
| Other | 6 | 1.8 |
|  |  |  |

Supplementary Table II: Results Study HoPE Fertility Quality of Life Questionnaire

|  | n  (%) | n  (%) | n  (%) | n  (%) | n  (%) | |
| --- | --- | --- | --- | --- | --- | --- |
|  | Very Poor | Poor | Neither Good nor Poor | Good | Very Good | |
| A: How would you rate your health? (n=304) | 3  (1.0) | 7  (2.3) | 37  (12.2) | 174  (57.2) | 83  (27.3) | |
|  | Very  Dissatisfied | Dissatisfied | Neither Satisfied nor Dissatisfied | Satisfied | Very  Satisfied | |
| B: Are you satisfied with your quality of life? (n=304) | 4  (1.3) | 25  (8.2) | 28  (9.2) | 199  (65.5) | 48  (15.8) | |
|  | Completely | A Great Deal | Moderately | Not Much | Not At All | |
| Q1: Are your attention and concentration impaired by thoughts of infertility? (n=304) | 17  (5.6) | 72  (23.7) | 120  (39.5) | 63  (20.7) | 32  (10.5) | |
| Q2: Do you think you cannot move ahead with other life goals and plans because of fertility problems? (n=304) | 28  (9.2) | 90  (29.6) | 93  (30.6) | 57  (18.8) | 36  (11.8) | |
| Q3: Do you feel drained or worn out because of fertility problems? (n=304) | 42  (13.8) | 93  (30.6) | 103  (33.9) | 35  (11.5) | 31  (10.2) | |
| Q4: Do you feel able to cope with your fertility problems? (n=304) | 32  (10.5) | 64  (21.1) | 146  (48.0) | 53  (17.4) | 9  (3.0) | |
|  | Very  Dissatisfied | Dissatisfied | Neither Satisfied nor Dissatisfied | Satisfied | Very  Satisfied | |
| Q5: Are you satisfied with the support you receive from friends with regard to your fertility problems? (n=303) | 17  (5.6) | 53  (17.5) | 100  (33.0) | 89  (29.4) | 44  (14.5) | |
| Q6: Are you satisfied with your sexual relationship even though you have fertility problems? (n=304) | 18  (5.9) | 58  (19.1) | 74  (24.3) | 119  (39.1) | 35  (11.5) | |
|  | Always | Very Often | Quite Often | Seldom | Never | |
| Q7: Do your fertility problems cause feelings of jealousy and resentment? (n=304) | 12  (3.9) | 95  (31.3) | 92  (30.3) | 97  (31.9) | 8  (2.6) | |
| Q8: Do you experience grief and/or feelings of loss about not being able to have a child (or more children)? (n=304) | 24  (7.9) | 105  (34.5) | 101  (33.2) | 59  (19.4) | 15  (4.9) | |
| Q9: Do you fluctuate between hope and despair because of fertility problems? (n=304) | 46  (15.1) | 123  (40.5) | 70  (23.0) | 48  (15.8) | 17  (5.6) | |
| Q10: Are you socially isolated because of fertility problems? (n=304) | 4  (1.3) | 40  (13.2) | 59  (19.4) | 129  (42.4) | 72  (23.7) | |
| Q11: Are you and your partner affectionate with each other even though you have fertility problems? (n=300) | 87  (29.0) | 96  (32.0) | 79  (26.3) | 37  (12.3) | 1  (0.3) | |
| Q12: Do your fertility problems interfere with your day-to-day work or obligations? (n=304) | 2  (0.7) | 24  (7.9) | 68  (22.4) | 151  (49.7) | 59  (19.4) | |
| Q13: Do you feel uncomfortable attending social situations like holidays and celebrations because of your fertility problems? (n=303) | 8  (2.6) | 35  (11.6) | 67  (22.1) | 133  (43.9) | 60  (19.8) | |
| Q14: Do you feel your family can understand what you are going through? (n=304) | 11  (3.6) | 40  (13.2) | 83  (27.3) | 125  (41.1) | 45  (14.8) | |
|  | An Extreme Amount | Very Much | A Moderate Amount | A Little | Not At All | |
| Q15: Have fertility problems strengthened your commitment to your partner? (n=298) | 52  (17.4) | 122  (40.9) | 75  (25.2) | 29  (9.7) | 20  (6.7) | |
| Q16: Do you feel sad and depressed about your fertility problems? (n=303) | 25  (8.3) | 110  (36.3) | 89  (29.4) | 63  (20.8) | 16  (5.3) | |
| Q17: Do your fertility problems make you inferior to people with children? (n=304) | 22  (7.2) | 68  (22.4) | 64  (21.1) | 67  (22.0) | 83  (27.3) | |
| Q18: Are you bothered by fatigue because of fertility problems? (n=303) | 36  (11.9) | 112  (37.0) | 67  (22.1) | 51  (16.8) | 37  (12.2) | |
| Q19: Have fertility problems had a negative impact on your relationship with your partner? (n=302) | 7  (2.3) | 34  (11.3) | 65  (21.5) | 97  (32.1) | 99  (32.8) | |
| Q20: Do you find it difficult to talk to your partner about your feelings related to infertility? (n=300) | 14  (4.7) | 28  (9.3) | 47  (15.7) | 72  (24.0) | 139  (46.3) | |
| Q21: Are you content with your relationship even though you have fertility problems? (n=299) | 91  (30.4) | 153  (51.2) | 47  (15.7) | 5  (1.7) | 3  (1.0) | |
| Q22: Do you feel social pressure on you to have (or have more) children? (n=304) | 36  (11.8) | 82  (27.0) | 71  (23.4) | 42  (13.8) | 73  (24.0) | |
| Q23: Do your fertility problems make you angry? (n=304) | 49  (16.1) | 80  (26.3) | 86  (28.3) | 54  (17.8) | 35  (11.5) | |
| Q24: Do you feel pain and physical discomfort because of your fertility problems? (n=304) | 12  (3.9) | 45  (14.8) | 68  (22.4) | 61  (20.1) | 118  (38.8) | |
| Optional treatment Module (n=110) | | | | | |  |
|  | Always | Very Often | Quite often | Seldom | Never | |
| T1: Does infertility treatment negatively affect your mood? (n=109) | 10  (9.2) | 28  (25.7) | 37  (33.9) | 32  (29.4) | 2  (1.8) | |
| T2: Are the fertility medical services you would like available to you? (n=106) | 16  (15.1) | 41  (38.7) | 31  (29.2) | 11  (10.4) | 7  (6.6) | |
|  | An Extreme Amount | Very Much | A Moderate Amount | A Little | Not At All | |
| T3: How complicated is dealing with the procedure and/ or administration of medication for your infertility treatment(s)? (n=109) | 10  (9.2) | 23  (21.1) | 42  (38.5) | 24  (22.0) | 10  (9.2) | |
| T4: Are you bothered by the effect of treatment on your daily or work- related activities? (n=108) | 10  (9.3) | 30  (27.8) | 41  (38.0) | 15  (13.9) | 12  (11.1) | |
| T5: Do you feel the fertility staff understand what you are going through? (n=108) | 13  (12.0) | 36  (33.3) | 41  (38.0) | 13  (12.0) | 5  (4.6) | |
| T6: Are you bothered by the physical side effects of fertility medications and treatment? (n=107) | 9  (8.4) | 29  (27.1) | 39  (36.4) | 17  (15.9) | 13  (12.1) | |
|  | Very  Dissatisfied | Dissatisfied | Neither Satisfied nor Dissatisfied | Satisfied | Very  Satisfied | |
| T7: Are you satisfied with the quality of services available to you to address your emotional needs? (n=109) | 14  (12.8) | 17  (15.6) | 36  (33.0) | 32  (29.4) | 10  (9.2) | |
| T8: How would you rate the surgery and/or medical treatment(s) you have received? (n=109) | 4  (3.7) | 11  (10.1) | 26  (23.9) | 50  (45.9) | 18  (16.5) | |
| T9: How would you rate the quality of information you received about medication, surgery and/or medical treatment? (n=109) | 4  (3.7) | 18  (16.5) | 23  (21.1) | 46  (42.2) | 18  (16.5) | |
| T10: Are you satisfied with your interactions with fertility medical staff? (n=109) | 3  (2.8) | 14  (12.8) | 18  (16.5) | 43  (39.4) | 31  (28.4) | |

Supplementary Table III: Results SpNQ-20 and Scoring.

| During the last time, did you have had the needs… | n (%) | n (%) | n (%) | n (%) |
| --- | --- | --- | --- | --- |
|  | No | Yes, somewhat | Yes, strong | Yes, very strong |
| To talk with others about your fears and worries? (n=279) | 48 (17.2) | 63 (22.6) | 110 (39.4) | 58 (20.8) |
| To plunge into beauty of nature? (n=279) | 36 (12.9) | 49 (17.6) | 99 (35.5) | 95 (34.1) |
| To dwell at a place of quietness and peace? (n=279) | 36 (12.9) | 47 (16.8) | 107 (38.4) | 89 (31.9) |
| To find inner peace? (n=279) | 47 (16.8) | 50 (17.9) | 79 (28.3) | 103 (36.9) |
| To dissolve / clarify open aspects of your life? (n=279) | 84 (30.1) | 65 (23.3) | 87 (31.2) | 43 (15.4) |
| To find meaning in illness and/or suffering? (n=277) | 88 (31.8) | 52 (18.8) | 73 (26.4) | 64 (23.1) |
| To talk with someone about the question of meaning in life? (n=279) | 154 (55.2) | 39 (14.0) | 58 (20.8) | 28 (10.0) |
| To talk with someone about the possibility of life after death? (n=279) | 215 (77.1) | 23 (8.2) | 26 (9.3) | 15 (5.4) |
| To forgive someone from a distinct period of your life? (n=279) | 185 (66.3) | 48 (17.2) | 27 (9.7) | 19 (6.8) |
| To be forgiven? (n=279) | 194 (69.5) | 35 (12.5) | 27 (9.7) | 23 (8.2) |
| To pray with someone? (n=278) | 233 (83.8) | 19 (6.8) | 14 (5.0) | 12 (4.3) |
| That someone prays for you? (n=278) | 194 (69.8) | 42 (15.1) | 23 (8.3) | 19 (6.8) |
| To pray for yourself? (n=278) | 186 (66.9) | 44 (15.8) | 32 (11.5) | 16 (5.8) |
| To participate at a religious ceremony (i.e. Sunday service)? (n=279) | 229 (82.1) | 34 (12.2) | 8 (2.9) | 8 (2.9) |
| To read religious / spiritual books? (n=278) | 229 (82.4) | 34 (12.2) | 8 (2.9) | 7 (2.5) |
| To turn to a higher presence (i.e., God, Allah, Angels, Saints)? (n=279) | 171 (61.3) | 53 (19.0) | 34 (12.2) | 21 (7.5) |
| To give away something from yourself? (n=278) | 177 (63.7) | 51 (18.3) | 35 (12.6) | 15 (5.4) |
| To give solace to someone? (n=279) | 109 (39.1) | 82 (29.4) | 57 (20.4) | 31 (11.1) |
| To pass own life experiences to others? (n=278) | 82 (29.5) | 62 (22.3) | 90 (32.4) | 44 (15.8) |
| To be assured that your life was meaningful and of value? (n=277) | 86 (31.0) | 52 (18.8) | 86 (31.0) | 53 (19.1) |
| Score: Needs for Inner Peace (n=279) | Mean:1.82, SD 0.77, Max:3, Min:0 | | | |
| Score: Existential Needs (n=279) | Mean:0.86, SD 0.71, Max:3, Min:0 | | | |
| Score: Religious Needs (n=279) | Mean:0.43, SD 0.65, Max:3, Min:0 | | | |
| Score: Giving/Generativity Needs (n=279) | Mean:1.09, SD 0.77, Max:3, Min:0 | | | |
| SpNQ20-Mean-Score (n=279) | Mean:0.97, SD 0.54, Max:3, Min:0 | | | |

SpNQ20 range = 0-3, whereof the highest score of 3 indicates a large need for psychosocial, existential, and spiritual, as well as for religious, existential, inner peace, and generative aspects
